# Immune Thrombocytopenic Purpura Following Pfizer-BioNTech COVID-19 Vaccine in an Elderly Female

**DOI:** 10.7759/cureus.16871

**Published:** 2021-08-04

**Authors:** Ranjit B Jasaraj, Dhan B Shrestha, Suman Gaire, Mohammed Kassem

**Affiliations:** 1 Department of Internal Medicine, Mount Sinai Hospital, Chicago, USA; 2 Department of Emergency Medicine, Palpa Hospital, Palpa, NPL; 3 Department of Hematology and Oncology, Mount Sinai Hospital, Chicago, USA

**Keywords:** covid-19 vaccine, mrna vaccines, pfizer-biontech, immune thrombocytopenia, covid-19

## Abstract

Mass vaccination campaigns are being run all over the globe to combat the ongoing COVID-19 pandemic. There have been several reports of immune thrombocytopenic purpura (ITP) occurrence following COVID-19 vaccination. However, ITP due to the Pfizer-BioNTech vaccine has been rarely reported, and a causal link has not been identified. The pathophysiology behind immune thrombocytopenia is similar to heparin-induced thrombocytopenia. The management is also similar to other secondary immune thrombocytopenia. We present a case of a 67-year old female diagnosed with immune thrombocytopenia following Pfizer-BioNTech vaccination. The treatment was resistant to high-dose steroids, intravenous immunoglobulin (IVIG), and rituximab and eventually responded to a thrombopoietin-stimulating agent.

## Introduction

Coronavirus disease-2019 (COVID-19) was declared a pandemic on March 11, 2020, by the WHO. As of July 10, 2021, there have been 33,604,986 total reported cases and 603,958 deaths in the United States [1]. Mass vaccination campaigns are being run all over the world to combat the pandemic. The U.S. Food and Drug Administration (US-FDA) has approved the Pfizer-BioNTech, Moderna, and Janssen COVID-19 vaccines, under Emergency Use Authorization (EUA). The US-FDA gave the Pfizer-BioNTech COVID-19 Vaccine EUA on December 11, 2020, for individuals 16 years or older. The EUA was expanded to include adolescents between 12 and 15 years on May 10, 2021 [2]. As of July 10, 2021, 184,469,317 Pfizer-BioNTech COVID-19 vaccines and a total of 332,966,409 vaccines have been administered in the US [1]. The Pfizer-BioNTech vaccine is an mRNA vaccine coated in a lipid that encodes the severe acute respiratory syndrome-coronavirus-2 (SARS-COV-2) spike glycoprotein.

Immune thrombocytopenic purpura (ITP) is an immune-mediated disease defined by isolated thrombocytopenia (peripheral blood platelet count < 100,000/uL) [3]. When other causes or diseases that have led to the condition are not identified, it is termed primary ITP. In contrast, secondary ITP refers to immune thrombocytopenia due to underlying conditions or drugs [3]. Various viral diseases like the human immunodeficiency virus (HIV), Varicella-Zoster virus, cytomegalovirus (CMV), Zika virus, and various autoimmune diseases like systemic Lupus Erythematosus have been associated with secondary ITP. The annual incidence of adult immune thrombocytopenia is around 3.3 per 100,000 in the US [4].

We present a case of a 67-year-old female who presented with ITP following the second dose of the Pfizer-BioNTech vaccine.

## Case presentation

A 67-year-old Hispanic female with a past medical history of hypertension, type 2 diabetes mellitus, hypothyroidism, depression, vitamin B12 deficiency, and chronic cluster headaches was referred to our hospital from the clinic after she was found to have low platelet levels. Past medical records have shown that she had a normal platelet count two months before presentation. She was under regular medication for the aforementioned comorbidities with no recent changes in the regimen.

**Figure 1:**
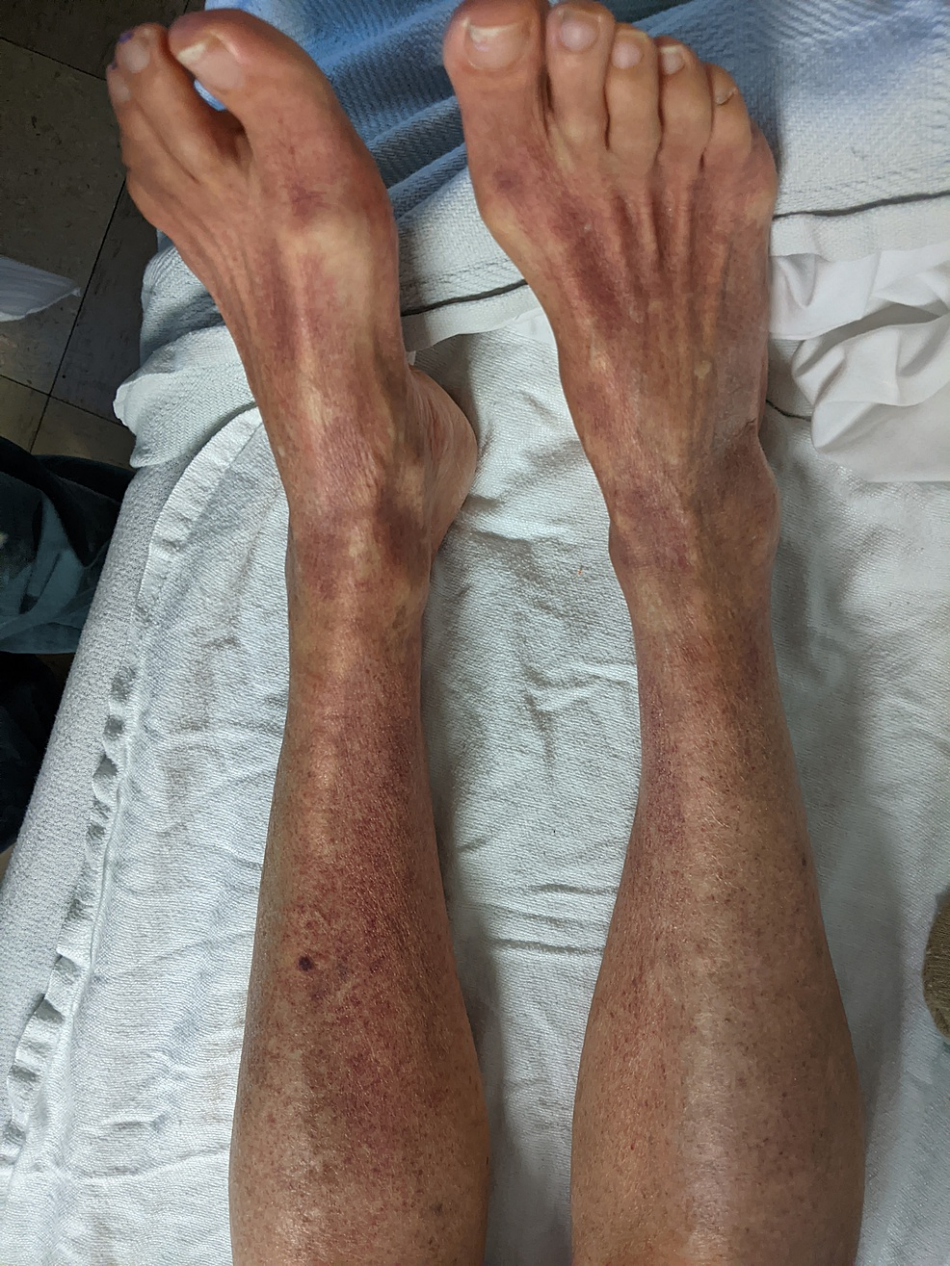
Ecchymosis over bilateral legs

**Figure 2:**
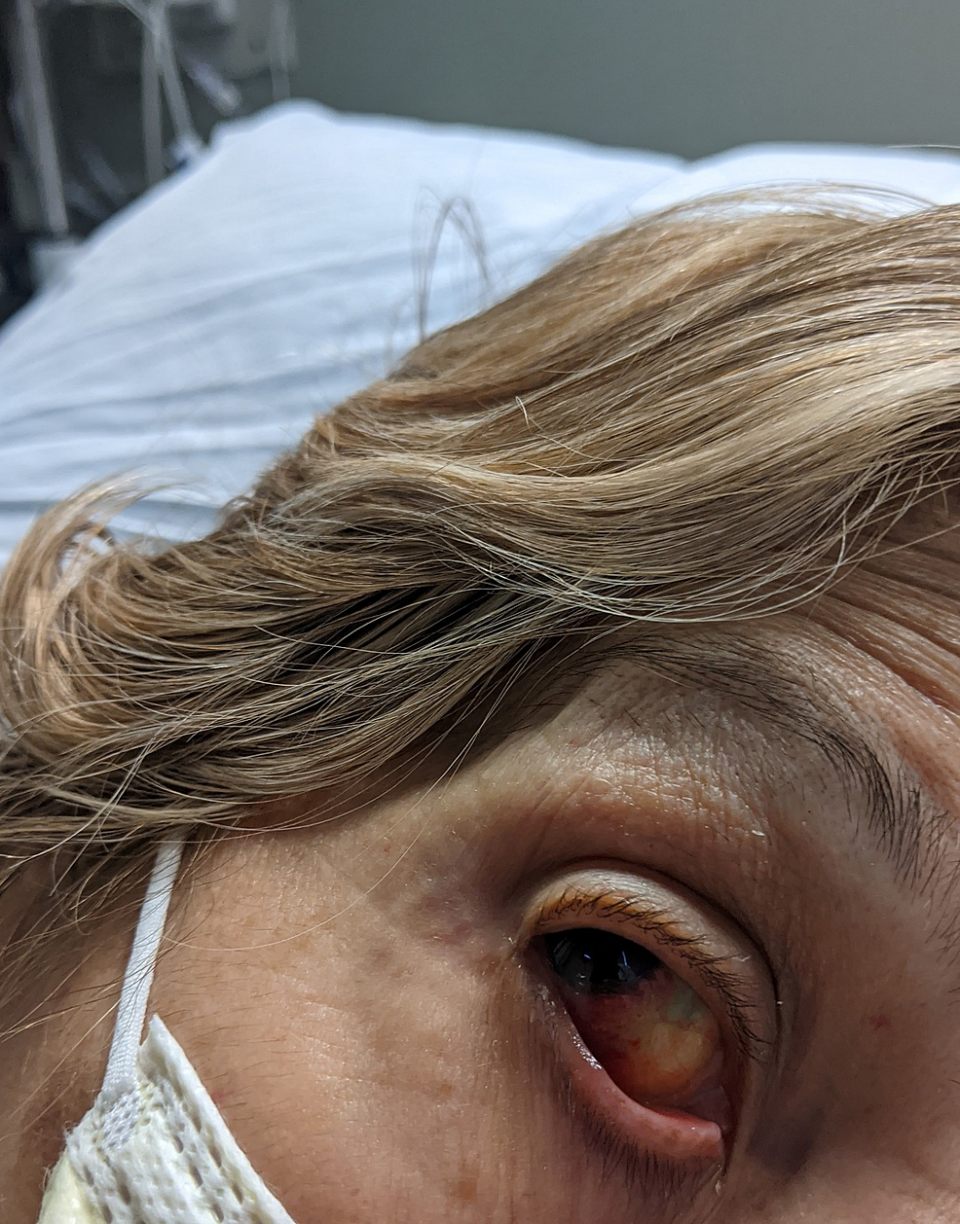
Subconjunctival hemorrhage

The patient had received the first dose of the Pfizer Bio-NTech COVID-19 vaccine about two months before hospitalization. She developed mild petechial rashes on her legs and chest after two weeks of the first dose of the vaccine. Rashes were insidious in onset and were non-pruritic. She denies any other adverse effects after her first dose of the vaccine. However, two days after her second dose of the vaccine, the patient noticed a rapid rash progression throughout her body without resolution of the rash (Figure 1). She also noticed bleeding in her gums when brushing her teeth and had an episode of epistaxis that resolved spontaneously. In addition, she developed a subconjunctival hemorrhage in the right eye (Figure 2) and hemorrhagic lesions of the tongue and buccal mucosa. On a subsequent clinic follow-up, her platelet count was found to be 3,000/µL, and she was referred to our hospital.

Upon admission, the patient complained of a constant right-sided headache. She did not have fever, chills, night sweats, vomiting, weight loss, lymphadenopathy, or any focal neurological deficits on presentation. She denied hemoptysis, hematemesis, melena, hematochezia, or hematuria. She reported a history of duodenal ulcers treated long back. Her vaccinations were up to date. She was diagnosed with hypertensive urgency, which was managed with antihypertensives. Laboratory investigations showed a hemoglobin level of 10.3 mg/dl and a platelet count of <3,000/µL. White blood cell count, coagulation studies, fibrinogen level, liver function tests (LFTs), lactate dehydrogenase (LDH), haptoglobin, and reticulocyte counts were normal. A CT scan of the head was negative for any acute intracranial hemorrhage. She tested negative for hepatitis B, C, HIV, Ebstein Bar virus, and CMV. She also tested negative for COVID-19. An ultrasound of the abdomen was negative for organomegaly.

The patient was immediately started on oral Prednisone at 1 mg/kg body weight and intravenous immunoglobulin (IVIG) at 400 mg/kg/day. However, her platelet counts did not improve, and she was transfused with 1 unit of platelets, given the high risk of bleeding. She was found to have a decline in her hemoglobin count, with her hemolytic lab panel being negative for hemolysis. Since she continued to develop large, ecchymotic patches on her arms, suggesting soft tissue bleed from each lab draw. She was started on aminocaproic acid at a loading dose of 5 g and continued on a maintenance dose of 1 g twice daily. She tolerated the treatment well, and her platelet level rose slowly to 10,000-15,000. She received three days of treatment with IVIG along with high-dose steroids, with her platelet count peaking at 10,000/µL. 

Upon further investigation, her autoimmune workup with anti-nuclear antibody (ANA), dsDNA, and complement levels was normal. Her rheumatoid factor was elevated to a level of 87 IU/mL, which eventually normalized. Her serum protein electrophoresis was normal. A bone marrow biopsy and aspirate were done, and pathology reported adequate megakaryocytes with trilineage hematopoiesis without evidence of myelodysplasia. She was discharged on day 14 with a platelet count of 6,000/µL. While closely following up in a Hematology/Oncology clinic, she received four doses of Rituximab. At this time, her petechial rashes and subconjunctival hemorrhage were starting to resolve. Since her platelet counts continued to stay below 10,000/µL, she was treated with a TPO agent (Eltrombopag). This eventually helped to increase her platelet count and maintain it over 200,000/µL over the next two months. Subsequently, her platelet counts have maintained above 200,000-250,000/fL, off of all medications.

## Discussion

We have presented a case of ITP occurrence following Pfizer-BioNTech vaccination. In the randomized, double-blinded, placebo-controlled trial of the Pfizer-BioNTech vaccine with 43,548 patients; only four severe adverse events were reported. The reported severe adverse events were shoulder injury related to vaccine administration, right axillary lymphadenopathy, paroxysmal ventricular arrhythmia, and right leg paresthesia. Additionally, two deaths were reported - one caused by arteriosclerosis and the other by cardiac arrest. All other adverse effects were mild [5].

Prior evidence has suggested that ITP occurrence may be seen after immunization with various vaccines. A direct, cause-effect relation between ITP and the measles mumps rubella (MMR) vaccine has been established [6]. Although a causal relationship has not been identified in many cases, ITP has been reported following several immunizations, including varicella live vaccine, human papilloma vaccine, *Hemophilus influenza* vaccine, hepatitis B, the polio vaccine, and the diphtheria-tetanus-pertussis vaccine [7].

Covid-19 may be associated with secondary ITP, which can be attributed to several mechanisms like immune dysregulation, molecular mimicry, expression of cryptic antigen on platelets, etc. [8]. Furthermore, thrombocytopenia may worsen in patients already suffering from chronic ITP following COVID-19 vaccine administration [9].

Several case reports of immune thrombocytopenia have been reported after the COVID-19 vaccination. In addition, reports have indicated vaccine-induced immune thrombocytopenia with the adenoviral vaccine, ChAdOx1, and the Janssen Covid-19 vaccine [10,11]. The pathogenesis behind vaccine-induced immune thrombocytopenia is similar to heparin-induced thrombocytopenia. Antibodies against platelet-activating factor 4 have been implicated, which causes massive platelet activation by widespread binding to the Fc receptor. This leads to platelet consumption with thrombosis and thrombocytopenia [10].

Cases of immune thrombocytopenia following the Pfizer Bio-NTech Covid-19 vaccine have not been as widely reported. There are reports of 35 possible cases of central nervous system thrombosis following vaccination [12] and two cases of deep vein thrombosis [13,14]. In addition, there have been some cases of thrombocytopenia following vaccination reported in the Vaccine Adverse Events Reporting System (VAERS). However, there are not enough cases to attribute to safety concerns following mRNA vaccines [15].

Vaccine-induced thrombocytopenia occurs 4 to 16 days after exposure to the vaccine [16]. However, in cases of earlier exposure to the immunogenic agent or the presence of naturally occurring antibodies, it can manifest earlier [17]. Our patient presented with ITP following the second dose of the Pfizer Bio-NTech Covid-19 vaccine. She also had developed some symptoms following the first dose. This could have caused a relatively earlier presentation in our patient.

IVIG and glucocorticoids are considered the first line in the management of ITP; accordingly, our patient was treated with. Our patient did not improve and had severe thrombocytopenia. She finally recovered after Rituximab and Eltrombopag therapies, which helped to raise her platelet counts. After three months of treatment, her platelet counts have stabilized, and she is off of all therapies.

## Conclusions

Immune thrombocytopenia occurrence after the Pfizer-BioNTech Covid-19 vaccine is rare and a causal relationship of the vaccine to ITP has not been established. In cases with prior exposure, ITP can be seen within hours to days of vaccine administration. We presented a case with severe thrombocytopenia, unresponsive to high-dose steroids, IVIG, and Rituximab. Subsequently, responding to thrombopoietin stimulating agent, Eltrombopag.
